# The Relationship and Effects of Self-Esteem and Body Shape on Eating Disorder Behavior: A Cross-Sectional Survey of Chinese University Students

**DOI:** 10.3390/healthcare12101034

**Published:** 2024-05-16

**Authors:** Zeng Gao, Jingyi Zhao, Sanying Peng, Han Yuan

**Affiliations:** 1 School of Educational Studies, Universiti Sains Malaysia, Pulau Pinang 11800, Malaysia; 2 Physical Education Department, Nanjing Institute of Technology, Nanjing 211167, China; 3 Physical Education Department, Hohai University, Nanjing 211100, China; 4 Department of Physical Education, Kyungpook National University, Daegu 41566, Republic of Korea

**Keywords:** somatotypes, self-perception, feeding and eating disorders, Chinese university students

## Abstract

Background: Eating disorders (EDs) have become a global public health concern among adolescents and young adults. However, Chinese university students exhibit a high prevalence of eating disorders. This study aims to investigate the effects of self-esteem (SE) and body shape (BS) on ED behaviors among Chinese university students. Methods: Using random sampling, 946 Chinese university students (aged 18 to 24, M = 19.94, SD = 1.04) participated in a survey comprising the Sick, Control, One, Fat, and Food Questionnaire (SCOFF-Q), the Body Shape Questionnaire (BS-Q), and the Rosenberg Self-Esteem Scale (RS-S) to assess their eating disorder or non-eating disorder (NED) behavior. Results: There was a significant positive correlation between body shape and eating disorder behaviors (r = 0.19, *p* < 0.01), while there was a significant negative correlation between self-esteem and eating disorder behaviors (r = −0.14, *p* = 0.001 < 0.01). Gender was a moderating factor in the relationship between body shape and eating disorder behaviors (t = 3.14, *p* = 0.002 < 0.01), while parents’ marital status was a moderating factor in the relationship between self-esteem and eating disorder behavior (t = 2.72, *p* = 0.007 < 0.01). Body shape (z = 6.47, *p* = 0.001 < 0.01), self-esteem (z = −2.81, *p* = 0.005 < 0.05), and gender (z = 3.06, *p* = 0.002 < 0.01) significantly influenced eating disorder behavior among Chinese university students aged 18–24 years. Conclusions: There was a direct effect between body shape and self-esteem and eating disorder behaviors among Chinese university students aged 18–24 years. Alarmingly, female university students are becoming susceptible to external influences on self-esteem and body shape, leading to eating disorder behaviors at an increasingly younger age in China.

## 1. Introduction

Eating disorders (EDs) have become one of the most prevalent health problems worldwide, described as risk-implying illnesses with psychiatric comorbidities in psychological and mental health, such as binge eating disorder (BED), bulimia nervosa (BN), anorexia nervosa (AN), and other illnesses, affecting both males and females during adolescence [1,2]. AN disorder mainly involves deliberate and sustained weight loss induced by the illness. In contrast, BN disorder is characterized by recurrent excessive preoccupation with BS and overeating, followed by compensatory behaviors to counteract perceived weight gain from the ingested food [3]. Both disorders are related to BS dissatisfaction and negative subjective evaluations of one’s body [4]. Individuals with EDs are at greater risk for poorer quality of life, suicide attempts, and death compared to individuals with other psychiatric disorders and the general population [5].

In the context of EDs, body shape is scrutinized through self-reports, notably in AN and BN, which are predominantly observed in young females [6]. This preoccupation becomes pronounced, serving as a core feature of severe EDs [7]. Severe AN has been associated with specific BS characteristics [8]. The *Diagnostic and Statistical Manual of Mental Disorders, Fifth Edition* (DSM-5) characterizes EDs by distorted perceptions of BS, weight, and pathological eating habits [9]. BS emerges as a tangible predictor of ED risk factors [10], particularly when dissatisfaction intensifies, accentuating ED behaviors [11]. So, the emotions that come with BS dissatisfaction further exacerbate ED behaviors. Appearance anxiety is significantly different from the prevalence of EDs (95% CI: 38.1–38.8%) in university students of different genders, with both females (SD = 39.21 ± 9.49, *p* < 0.01) and males (SD = 38 ± 9.42, *p* < 0.01) displaying significant differences [12]. This also confirms the correlation between BS and EDs.

Self-esteem (SE) is defined as a relatively stable trait reflecting an individual’s overall values and self-perception [13]. Mora et al. [14] reported a correlation between SE and EDs. Males constitute 25% of cases of AN and BN within EDs [15]. Despite the recognized link between ED pathology and SE, empirical evidence on the developmental aspects of SE concerning problematic eating patterns and eating disorder-related conditions, considering other variables in the model, remains limited [16,17,18]. Additionally, SE has been proven to be an ED risk factor among individuals with BN and AN [19,20]. Positive SE is associated with symptoms of BN and AN, which can affect EDs [21,22]. This further illustrates the interaction between BS, EDs, and SE [21].

The symptoms of EDs are prevalent among adolescents, constituting a global public health issue affecting populations aged 13 to 25/30, including both males and females, notably among university students [12,23,24]. Epidemiological studies in Australia and The Netherlands have consistently reported a high prevalence of ED behaviors, ranging from 14% to 22%, within young adult populations [23,24,25]. According to the University of Delhi in India, a study proved a correlation between BS, SE, and EDs among female undergraduate students [26]. In previous studies, Guan and Wang [27] reported a prevalence rate of 2.5% for female university students in Beijing, Fu et al. [28] found a prevalence rate of 2.8% among female university students, Li et al. [29] identified prevalence rates of 6.50% for males and 14.3% for females, and Yu et al. [30] observed prevalence rates of 5.3% for males and 4.0% for females in Anhui Province. The latest survey conducted in China revealed a striking rate of 38% (CI: 38.1–38.8%, 95%) of university students suffering from EDs, with notable gender differences between males (CI: 30.7–31.6%, 95%) and females (CI: 43.2–44.0%, 95%) [12]. Therefore, the phenomenon is on the rise among Chinese university students. Additionally, female young adults, both within China and internationally, exhibit a heightened prevalence of ED risks between the ages of 18 and 25, with symptoms often accompanied by elevated body mass index (BMI) levels (*p* < 0.01), leading to overweight and obesity, objective BED, and compensatory behaviors [31]. Eating attitudes (2.5%) are more prevalent than actual EDs (0.90%) among Chinese university students, with females (3.2%) experiencing a significantly higher prevalence than males (1.2%) [31].

**Figure 1:**
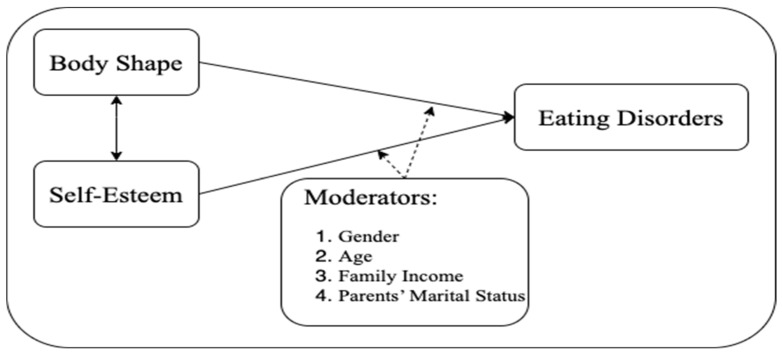
Conceptual framework.

The increasing prevalence of ED behaviors among Chinese university students underscores the need for extensive research to elucidate high-risk factors, facilitate early identification in this population, and effectively predict the risk of ED behaviors. Therefore, this study aims to explore the joint predictive role of BS and SE in determining ED behaviors among Chinese university students. The study utilizes BS and SE as independent variables, with EDs as the dependent variable, and introduces additional variables, namely gender, age, family income, and parents’ marital status, as moderators to evaluate their interrelationships. The hypothesized model is visually represented in Figure 1.

## 2. Methods

### 2.1. Research Hypotheses

The research hypotheses are below:

**H1.**  *Demographic variables and eating disorders have significant differences*.

**H2.**  *Body shape, self-esteem, and eating disorders have significant relationships*.

**H3.**  *Gender, age, family income, and parents’ marital status have significant moderation effects on body shape and self-esteem to eating disorders*.

**H4.**  *Body shape, self-esteem, gender, age, family income, and parents’ marital status have significant predictive effects on eating disorders*.

### 2.2. Participants

The project employed a random sampling technique to gather an initial sample of 962 participants, including 16 (1.66%) participants who were excluded from the final analyses. A total of 946 participants (98.34%) were included in the analysis of Chinese university students aged 18 to 24 years. The study data were collected online between 1 September and 20 September 2023. The age range of the participants was determined based on the responses provided in their questionnaires. We utilized G*Power 3.1 software (effect size f^2^ of 0.05, α err prob of 0.01, power (1-β err prob) of 0.99) to calculate the sample size of 738. At the same time, in the Mayerhofer et al. [32] survey, 913 respondents were included in the analysis, and the Torstveit et al. [33] survey of 938 respondents further confirmed this study’s reasonable sample size. In addition, all variables’ skewness coefficients and the kurtosis coefficients were both less than 1, indicating a normal distribution. Before conducting the survey, we ensured that all participants were fully informed about the purpose of the research, its procedures, potential benefits, and risks. Responses were anonymous, and participants could withdraw at any time, and the study involved only non-sensitive questions and assured the anonymity and confidentiality of the respondents. The study did not pose any potential risks or adverse effects to the participants. Before completing the survey, participants read and consented to the study’s aim of volunteering without compensation. The procedure was under the guidelines provided by Universiti Sains Malaysia. In addition, demographic characteristics, including gender, parents’ marital status, and variations in family income groups, were self-reported by Chinese university students.

### 2.3. Instruments

#### 2.3.1. The SCOFF-Q

The SCOFF-Q Chinese Version, consisting of five items, was utilized to assess ED behavior, specifically AN or BN [34]. Each item was rated on a 2-point Likert scale (0 = “no” and 1 = “yes”). A score of 2 or more indicated potential ED behavior. The Chinese version demonstrated a moderate test–retest reliability of 0.66 (95% CI, 0.276–0.838) [34].

#### 2.3.2. The BS-Q

The Chinese version of the Body Shape Questionnaire (BS-Q) with eight items was employed to evaluate body shape concerns related to EDs [35]. Respondents used a 6-point Likert scale (“Never—1” to “Always—6”). The questionnaire demonstrated high reliability, with a coefficient of 0.94 [35,36].

#### 2.3.3. The RS-S

The Chinese version of the Rosenberg Self-Esteem Scale (RS-S) included ten items to assess self-esteem issues [37]. Each item was rated on a 4-point Likert scale (“Strongly Disagree—1” to “Strongly Agree—4”). The questionnaire exhibited strong internal consistency, with reliability coefficients ranging from 0.76 to 0.79 among Chinese students [37]. The RS-S consisted of ten items, of which six were positive (Items 1, 2, 4, 6, 7, and 8) and four were negative (Items 3, 5, 9, and 10) [38].

### 2.4. Statistical Analysis

The study adopted SPSS version 24.0 software to analyze the data using descriptive statistics, Pearson correlation analysis, moderation analysis, and logistic regression analysis [39]. All variables conformed to the normal distribution. Descriptive statistics were used to calculate the frequency, percentage, and chi-squared test of categorical variables and the t-test of continuous variables. The Pearson correlation analysis utilized *p*-values to confirm the correlation between self-esteem, body shape, and EDs using continuous variable analysis. The moderating analysis was examined with gender, age, family income, and parents’ marital status as moderating variables to assess their influence on the relationships between EDs and BS, as well as EDs and SE, using analysis of continuous variables through the SCOFF-Q, BS-Q, and RS-S. The logistic regression analysis predicted the effects of BS, SE, gender, age, family income, and parents’ marital status on EDs. All questionnaires utilized in this study were the Chinese versions published by previous researchers. This approach aimed to mitigate cultural background differences and ensure consistency in data collection methods. Multivariate analysis was performed using logistic regression analysis, with the ED questionnaire scores as the dependent variables and SE and BS as the independent variables using categorical variables analysis.

## 3. Results

### 3.1. Reliability and Validity

The data were checked for reliability and validity by Cronbach’s alpha values, factor loading, and KMO values (in Table 1). All items had factor loadings over 0.5, and all Cronbach’s alpha values were more than 0.60, with KMO values over 0.70. This further showed that all the questionnaire values and structures were good among Chinese university students.

### 3.2. Descriptive Statistics

The chi-squared test was utilized to analyze the categorical variables among the three groups of EDs, aiming to determine if there was a statistically significant difference in the demographic variables. As shown in Table 2, the average ages of all participants, No-ED, and Yes-ED, were 19.98, 20.06, and 19.89 years old. The chi-squared test results showed significant differences (*p* < 0.05) in demographic variables between SE (*χ^2^* = 16.618, *p* = 0.001 < 0.01), BS (*χ^2^* = 54.870, *p* = 0.001 < 0.01), gender (*χ^2^* = 10.442, *p* = 0.001 < 0.01), and family income (*χ^2^* = 6.026, *p* = 0.049 < 0.05) and EDs. Additionally, significant difference analyses were conducted to determine if gender, age, family income, and parents’ marital status, as independent demographic variables, had significant differences from all the independent variables and the dependent variable. Due to differences in family income, we divided the family income into different level groups, namely low (below 16,443 RMB/year), average (between 16,443 and 41,172 RMB/year), and good (above 41,172 RMB/year). We used the different groups of family incomes (continuous variables) as categorical variables for analysis.

### 3.3. Pearson Correlation Analysis

Table 3 presents the Pearson correlation coefficients, analyzing the correlations between BS, SE, and EDs. The correlation coefficient value between EDs and BS (r = 0.19, *p* =0.001 < 0.01) indicated a significant positive correlation between EDs and BS. It further proved that there were statistically significant relationships between the different BS scores and their sub-categories (from no concern with shape to mild concern with shape to moderate concern with shape to marked concern with shape) with ED behaviors (*p* < 0.05). Moreover, the correlation coefficient value between EDs and SE (r = −0.14, *p* = 0.001 < 0.01) showed a significant negative correlation between EDs and SE. It further proved that there were statistically significant relationships between the different SE scores and their sub-categories (from low self-esteem to normal self-esteem to high self-esteem) and ED behaviors (*p* < 0.05). Additionally, we also found that BS and SE showed a significant negative correlation (r = −0.39, *p* = 0.001< 0.01). It further proved that there were statistically significant relationships between BS scores and its sub-categories (from no concern with shape to mild concern with shape to moderate concern with shape to marked concern with shape) and SE scores and its sub-categories (from low self-esteem to normal self-esteem to high self-esteem) (*p* < 0.05).

### 3.4. Moderating Variables of BS and SE with EDs

Table 4 shows that gender had a significant effect as a moderator variable on the relationship between BS and EDs (t = 3.14, *p* = 0.002 < 0.01). Age had no significant effect as a moderator variable on the relationship between BS and EDs (t = −0.68, *p* = 0.498 > 0.05). Family income had no significant effect as a moderator variable on the relationship between BS and EDs (t = 0.40, *p* = 0.692 > 0.05). Parents’ marital status had no significant effect as a moderator variable on the relationship between BS and EDs (t = −1.76, *p* = 0.080 > 0.05). However, parents’ marital status had a significant effect as a moderator variable on the relationship between SE and EDs (t = 2.72, *p* = 0.007 < 0.01). Gender had no significant effect as a moderator variable on the relationship between SE and EDs (t = 0.82, *p* = 0.414 > 0.05). Age had no significant effect as a moderator variable on the relationship between SE and EDs (t = −0.63, *p* = 0.532 > 0.05). Family income had no significant effect as a moderator variable on the relationship between SE and EDs (t = 0.50, *p* = 0.620 > 0.05). Therefore, gender and parents’ marital status have significant moderating effects on BS and SE in EDs.

### 3.5. Predicting the Impact of All Variables on EDs

Table 5 presents the logistic regression analysis. The initial hypothesis assumed that the model comprising BS, SE, gender, age, family income status, and parents’ marital status was invalid. However, the rejection of this hypothesis and the subsequent indication of the model’s effectiveness (chi-square = 74.24, *p* = 0.001 < 0.01) provided compelling evidence. Utilizing BS, SE, gender, age, family income status, and parents’ marital status as independent variables, the analysis aimed to predict EDs as the dependent variable.

**Figure 2:**
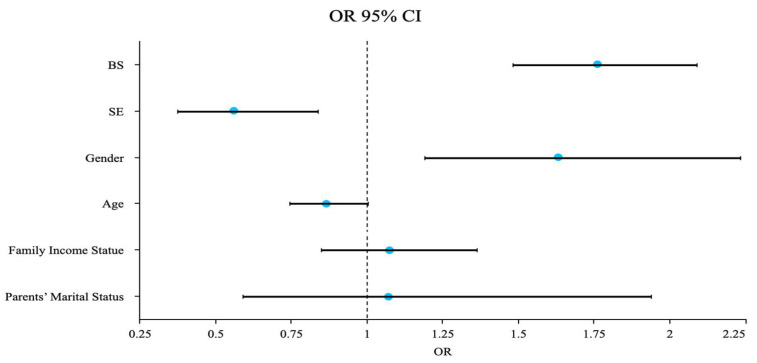
OR coefficient plot.

In Table 5 and Figure 2, the results reveal that BS exerted a significant positive impact on EDs (z = 6.47, *p* = 0.000 < 0.01), with the prevalence of EDs increasing by 1.76 times (OR = 1.76) for each additional category of BS. Conversely, SE exhibited a significant negative impact on EDs (z = −2.81, *p* = 0.005 < 0.01), leading to a decrease in the prevalence of EDs by 0.56 times (OR = 0.56) for each additional category of SE. Moreover, gender demonstrated a significant positive association with EDs (z = 3.06, *p* = 0.002 < 0.01), with females showing a prevalence of EDs of 1.63 times higher than males (OR = 1.63). However, age (z = −1.910, *p* = 0.056 > 0.05), family income status (z = 0.594, *p* = 0.553 > 0.05), and parents’ marital status (z = 0.223, *p* = 0.823 > 0.05) were found to have no significant impact on EDs (*p* > 0.05). Therefore, these results proved that BS and gender have a significant positive effect on EDs, SE has a significant negative impact on EDs, and age, family income status, and parents’ marital status have no impact on EDs.

## 4. Discussion

This study evaluated the relationship and effects of BS and SE (independent variables) on ED behaviors (dependent variable) among Chinese university students. We examined the moderating roles of gender, age, family income, and parents’ marital status. The findings revealed that (1) BS, SE, gender, and family income have significant differences among Chinese university students aged 18–24 years; (2) BS, SE, and EDs have significant relationships among Chinese university students aged 18–24 years; (3) gender and parents’ marital status in terms of body shape and self-esteem in terms of eating disorders have significant moderation effects among Chinese university students aged 18–24 years; and (4) BS, SE, and gender have significant predictive effects on EDs among Chinese university students aged 18–24 years. These findings also underscore that the prevalence of EDs in China may be underestimated compared to Western countries worldwide [40], and BS, SE, and other variables have a relationship and be able to be predicted. Several studies have explored the prevalence of EDs in China and have also identified adolescents or young adults as target populations for research and prevention efforts [41,42,43].

Previous studies have identified BS and SE as risk factors for EDs (including AN, BN, BED, and others) across different genders, ages, family income levels, and marital statuses of parents in Western countries [2,8,10,19,20]. Studies have also reported a high prevalence of ED behaviors among individuals of different genders, and varying demographic characteristics pose a risk to physical and mental health in China [12,23,25,31].

However, a high prevalence of ED behaviors leads to risk-implying illnesses with psychiatric comorbidities in physical and mental health [1,2]. In addition, the findings from the survey revealed a heightened prevalence of BS contributing to eating disorder symptoms, especially precipitating appearance anxiety and social anxiety disorder, which led to poor physical and mental health among Chinese university students [12]. ED symptoms are also often accompanied by elevated body mass index (BMI) levels (*p* < 0.01), leading to overweight and obesity, objective BED, and compensatory behaviors [31]. The sampled male and female participants corroborated these findings regarding BS and EDs, aligning with studies conducted in different countries [44,45]. Furthermore, studies have underscored a higher prevalence of ED behaviors related to BS among male and female young adults aged 13 to 30 years in diverse countries [12,23,24]. This corroborated the high prevalence of 14–22% between BS and ED behaviors reported in epidemiological studies [23,25]. Additionally, one study unearthed a robust association between BS and severe AN [8]. The study’s results affirmed BS as a predictor of ED behaviors, particularly when considered as an independent risk factor [10].

A recent study investigated interventions aimed at promoting intuitive eating among Chinese females experiencing BS dissatisfaction and EDs, revealing that such interventions significantly improved BS and EDs [46]. Moreover, substantial cultural disparities exist in BS satisfaction and EDs between Chinese females and non-Hispanic white females, with factors like dairy restraint being notable contributors [47]. Additionally, the efficacy of clinical interventions for EDs is influenced by cultural background. Early interventions targeting BS and EDs among young Chinese females, along with their family intervention model, indicate that cognitive behavioral therapy and dialectical behavior therapy can effectively manage EDs in this population, despite their prolonged duration and associated costs [48]. So, this further provides new clues to the pathogenesis, risk factors, and intervention strategies of eating disorders. At the same time, it equips healthcare professionals with a wide range of treatment modalities for identifying and managing eating disorders, spanning from psychotherapy and nutritional counseling to medication.

Moreover, this study highlighted a correlated relationship (r = −0.15) between SE and EDs, which is consistent with the results reported by Mora et al. [14], and substantiated significant differences between SE and EDs (*p* < 0.05). Previous studies utilizing the Rosenberg Self-Esteem Scale and the Eating Attitudes Test-26 further supported the correlation between SE and EDs in youth [14,49]. However, in the context of SE as an independent variable, Ayala [19] posited that SE could not predict ED risk factors, aligning with the current study’s findings (*p* = 0.23). Longitudinal studies consistently indicate that women residing in urban areas exhibit a preference for thinner bodies and tend to have lower levels of body-related SE [50], rendering them more vulnerable to EDs. Furthermore, social norms not only vary across cultures, social classes, races, and genders but also evolve, leading to significant shifts in the epidemiology of body preferences and EDs [51]. Among ethnic cultures, it has been demonstrated that perceived maternal criticism is strongly linked to EDs among Asian Americans, particularly concerning their self-perception orientation [52].

Gender emerged as a pivotal moderator, revealing a significant relationship between BS, SE, and EDs. The analysis of ED behaviors by gender indicated a higher proportion of males suffering from AN and BN within the spectrum of EDs [15]. The phenomenon of university or college students regulating their daily meals based on BS to mitigate concerns about body dissatisfaction and the subsequent onset of ED behaviors has been elucidated [11]. It underscores the prevalence of body appearance anxiety, particularly among university students of both genders [12]. According to this study, we can provide help to clinical practice and public health policymaking by improving the living ways of patients or students with eating disorders and reducing related social health problems.

Several limitations should be acknowledged when interpreting the results of this study. Firstly, there may have been minor errors in the study findings for Chinese university students [53]. Additionally, reliance on self-reported data may contribute to potential biases, as respondents’ responses and behaviors are influenced by their ability to accurately recall, and report information related to eating disorders [54]. To address these limitations and enhance the robustness of future studies, increasing the sample size should be considered. Furthermore, while the use of questionnaires is common in studies, the absence of clinical interviews for a definitive diagnosis limits the precision of the prevalence figures [55]. Despite these constraints, the study’s findings provide valuable insights into the thinking associated with eating disorder behaviors among Chinese university students, emphasizing the importance of addressing this issue.

## 5. Conclusions

In summary, this study proved a relationship in the three-factor interaction model between body shape, self-esteem, and eating disorder behavior among Chinese university students aged 18–24 years. These results suggest that BS, SE, gender, and age have a relationship and effect on ED behavior, especially among the youth of female university students who are more susceptible to outside influences on SE and BS and are suffering from ED behaviors in China. Therefore, it is necessary to establish correct perceptions of BS and good SE to promote psychological stability and mental health among university students. Furthermore, future research should explore the complex interplay and effects of psychological factors on ED behaviors across various disease populations while also enhancing prevention efforts within educational and public health settings. And it should further distinguish different types of ED behaviors (such as AN, BN, and BED) from individual differences and contextual influences. Simultaneously, there is a need to enhance the dissemination of knowledge and curriculum-based education on healthy BS and SE and EDs among Chinese university students, foster a healthy public environment, aim to reduce the prevalence of EDs, and further investigate the underlying determinants identified in this study.

## Figures and Tables

**Table 1:** Reliability and validity.

Variable	Items	Factor Loading	Cronbach α	KMO
BS	BS1	0.73	0.93	0.93
	BS2	0.79		
	BS3	0.84		
	BS4	0.85		
	BS5	0.87		
	BS6	0.86		
	BS7	0.85		
	BS8	0.82		
SE	SE1	0.68	0.68	0.79
	SE2	0.76		
	SE3	0.72		
	SE4	0.79		
	SE5	0.83		
	SE6	0.84		
	SE7	0.63		
	SE8	0.68		
	SE9	0.85		
	SE10	0.72		
EDs	ED1	0.62	0.61	0.73
	ED2	0.72		
	ED3	0.92		
	ED4	0.75		
	ED5	0.58		

**Table 2:** Demographic characteristics and descriptive statistics.

Item	Categories	All Participants (*n* = 946) *n* (%)	No-ED (*n* = 704) *n* (%)	Yes-ED (*n* = 242) *n* (%)	*χ^2^*	*p*
Gender	Male	460 (48.63)	364 (51.70)	96 (39.67)	10.442	0.001 **
	Female	486 (51.37)	340 (48.30)	146 (60.33)		
Age	18 yrs.	60 (6.34)	39 (5.54)	21 (8.68)	9.799	0.133
	19 yrs.	216 (22.83)	150 (21.31)	66 (27.27)		
	20 yrs.	407 (43.02)	314 (44.60)	93 (38.43)		
	21 yrs.	196 (20.72)	148 (21.02)	48 (19.83)		
	22 yrs.	48 (5.07)	39 (5.54)	9 (3.72)		
	23 yrs.	16 (1.69)	11 (1.56)	5 (2.07)		
	24 yrs.	3 (0.32)	3 (0.43)	0		
Average Age		20.02 ± 1.05	20.06 ± 1.04	19.89 ± 1.07		
Family Income	Low (below 16,443 RMB/year)	418 (44.19)	304 (43.18)	114 (47.11)	6.026	0.049 *
	Average (between 16,443 and 41,172 RMB/year)	435 (45.98)	338 (48.01)	97 (40.08)		
	Good (above 41,172 RMB/year)	93 (9.83)	62 (8.81)	31 (12.81)		
Parents’ Marital Status	Married	885 (93.55)	662 (94.03)	223 (92.15)	1.061	0.303
	Divorced/Widowed	61 (6.45)	42 (5.97)	19 (7.85)		
BS	NO-CS	184 (19.45)	160 (22.73)	24 (9.92)	54.870	0.001 **
	MI-CS	362 (38.27)	285 (40.48)	77 (31.82)		
	MO-CS	289 (30.55)	203 (28.84)	86 (35.54)		
	MA-CS	111 (11.73)	56 (7.95)	55 (22.73)		
SE	L-SE	112 (11.84)	66 (9.38)	46 (19.01)	16.618	0.001 **
	N-SE	810 (85.62)	618 (87.78)	192 (79.34)		
	H-SE	24 (2.54)	20 (2.84)	4 (1.65)		

Notes: NO-CS is no concern with shape; MI-CS is mild concern with shape; MO-CS is moderate concern with shape; MA-CS is marked concern with shape; L-SE is low self-esteem; N-SE is normal self-esteem; H-SE is high self-esteem; * p < 0.05; ** *p <* 0.01.

**Table 3:** Pearson correlation analysis.

Item		ED	BS	SE
ED	*r*	1		
	*p*	-		
BS	*r*	0.19 **	1	
	*p*	0.001	-	
SE	*r*	−0.14 **	−0.39 **	1
	*p*	0.001	0.001	-

Note: ** *p* < 0.01.

**Table 4:** Moderation effects of BS and SE on EDs.

Variable	BS→EDs					SE→EDs				
	*B*	*S.E.*	*t*	*p*	*β*	*B*	*S.E.*	*t*	*p*	*β*
Gender	0.05	0.02	3.14	0.002 **	0.10	0.04	0.05	0.82	0.414	0.03
Age	−0.01	0.01	−0.68	0.498	−0.02	−0.01	0.02	−0.63	0.532	−0.02
Family Income	0.01	0.01	0.40	0.692	0.01	0.02	0.04	0.50	0.620	0.02
Parents’ Marital Status	−0.07	0.04	−1.76	0.080	−0.06	0.35	0.13	2.72	0.007 **	0.09

Notes: dependent variable: EDs; ** *p* < 0.01; *B*: unstandardized regression coefficient, *S.E.*: standard error, *β*: standardized regression coefficient.

**Table 5:** Logistic regression analysis.

Variable	*z* Value	Wald χ^2^	*p*	*OR*	*OR* 95% CI
BS	6.47	41.79	0.001 **	1.76	1.48~2.09
SE	−2.81	7.87	0.005 **	0.56	0.37~0.84
Gender	3.06	9.35	0.002 **	1.63	1.19~2.24
Age	−1.91	3.65	0.056	0.87	0.75~1.00
Family Income Status	0.59	0.35	0.553	1.08	0.85~1.36
Parents’ Marital Status	0.22	0.12	0.823	1.07	0.59~1.94

Notes: dependent variable: EDs, McFadden R Square: 0.069; Cox and Snell R Square: 0.075; Nagelkerke R Square: 0.111; ** *p* < 0.01.

## Data Availability

Data are contained within the article.

## Abbreviations

BS	body shape
EDs	eating disorders
NEDs	non-eating disorders
SE	self-esteem
SCOFF-Q	Sick, Control, One, Fat, and Food Questionnaire
BS-Q	Body Shape Questionnaire
RS-S	Rosenberg Self-Esteem Scale
NO-CS	no concern with shape
MI-CS	mild concern with shape
MO-CS	moderate concern with shape
MA-CS	marked concern with shape
L-SE	low self-esteem
N-SE	normal self-esteem
H-SE	high self-esteem
